# Herbal Products and Their Active Constituents for Diabetic Wound Healing—Preclinical and Clinical Studies: A Systematic Review

**DOI:** 10.3390/pharmaceutics15010281

**Published:** 2023-01-14

**Authors:** Anna Herman, Andrzej Przemysław Herman

**Affiliations:** 1 Chair of Drug and Cosmetics Biotechnology, Faculty of Chemistry, Warsaw University of Technology, Koszykowa 75 Street, 00-662 Warsaw, Poland; 2 Department of Genetic Engineering, The Kielanowski Institute of Animal Physiology and Nutrition, Polish Academy of Sciences, Instytucka 3 Street, 05-110 Jabłonna, Poland

**Keywords:** herbal products, diabetic wounds, foot ulcer wounds, bacterial diabetic wound infections, diabetic wound dressing

## Abstract

The purpose of this review is to provide verified data on the current knowledge acquired in preclinical and clinical studies regarding topically used herbal products and their active constituents (formulations and dressings) with diabetic wound healing activity. Moreover, herbal products and their active constituents used for diabetic wound infections, and various cellular and molecular mechanisms of their actions will also be described. The electronic databases were searched for articles published from 2012 to 2022. Publications with oral or systemic administration of herbal products in diabetic wound healing, published before 2012, available only as an abstract, or in languages other than English were excluded from the study. The 59 articles comparing topically used herbal products in diabetic wound healing treatment versus control treatments (placebo or active therapy) were selected. Herbal products through different mechanisms of action, including antimicrobial, anti-inflammatory, antioxidant activity, stimulation of angiogenesis, production of cytokines and growth factors, keratinocytes, and fibroblast migration and proliferation may be considered as an important support during conventional therapy or even as a substitute for synthetic drugs used for diabetic wound treatment.

## 1. Introduction

Diabetes is a metabolic disorder associated with the endocrine system that resulted in hyperglycemic conditions. Prolonged and untreated hyperglycemia or uncontrolled glucose levels leads to serious diabetic complications, such as nephropathy, neuropathy, retinopathy, hypertension, hyperlipidemia, increased risk of cardiovascular disease, and heart attacks [1]. The most costly and devastating complication of diabetes is delayed wound healing processes which can lead to serious complications such as a high risk of bacterial infections, gangrene, limb amputations, sepsis, and even death [2]. About 15% of diabetic patients have diabetic foot ulcers (DFU), and 14–24% of these patients subsequently experience a lower extremity amputation, with the mortality rate from amputation approaching 50–59% five years post-amputation [3]. Therefore, acceleration of wound healing should be a priority in preventing diabetes complications.

Wound healing difficulties in diabetes patients are multidirectional. The hyperglycemic condition in the wound site leads to chronic inflammation, impaired vascularization and tissue regeneration, reduced production of growth factors, excessive protease activity, and oxidative stress [4]. Moreover, open wounds are particularly prone to infection, especially by bacteria, and provide an entry point for systemic infections. Aerobic or facultative pathogens such as *Staphylococcus aureus*, *Pseudomonas aeruginosa*, and β-hemolytic streptococci are the primary causes of delayed healing and infection in both acute and chronic wounds [5,6]. Therefore, an important part of the standard treatment of diabetic wounds, in addition to wound debridement, revascularization, and acceleration of the healing process, is the control of bacterial infections. Topical antimicrobial agents (silver nitrate, povidone-iodine) or systemic administration of antibiotics (silver sulfadiazine, mafenide, mupirocin, bacitracin) do not always help to reduce the risk of progressive infection, especially if the bacteria are antibiotic resistant [7]. The reduction in wound healing time is crucial for diabetes especially to lower the chance of infection and decrease complications and costs. Herbal products and their active compounds may inhibit bacterial growth and may have a significant clinical value in the treatment of resistant microbial strains [8]. Moreover, some herbal products affect wound healing activities through anti-inflammatory and antioxidant activities, cell proliferation, and angiogenesis [9,10]. The purpose of this review is to provide verified data on the current knowledge acquired in preclinical and clinical studies regarding topically used herbal products (formulations and dressings) with diabetic wound healing activity. Moreover, herbal products and their active constituents used for microbial diabetic wound infections, and the various cellular and molecular mechanisms of their actions will also be described.

## 2. Methods

### 2.1. Search Strategy

This review was conducted and narrated in accordance with the Preferred Reported Items for Systematic Review and Meta-analysis (PRISMA) guidelines [11]. The PubMed, Scopus, and Google Scholar databases were searched for articles published from 2012 to 2022. Search terms included “herbal products and diabetic wound healing”, “herbal products and diabetic foot ulcers”, “plant extracts used in diabetic wounds”, “essential oils used in diabetic wounds”, and “herbal constituent used in diabetic wounds”. The references from reviews about herbal products and topical treatment of diabetic wounds were searched for additional articles and case reports. A manual search was also conducted based on citations in the published literature.

### 2.2. Inclusion and Exclusion Criteria

The results of animal- and human-based studies on topically used herbal products in diabetic wound healing comparing herbal products treatment versus control treatments (placebo or active therapy) were selected. Routes of administration other than topical (e.g., oral, systemic) of herbal products in diabetic wound healing were excluded from the study. Moreover, publications published before 2012, available only as an abstract, or in languages other than English were excluded.

### 2.3. Study Selection

**Figure 1:**
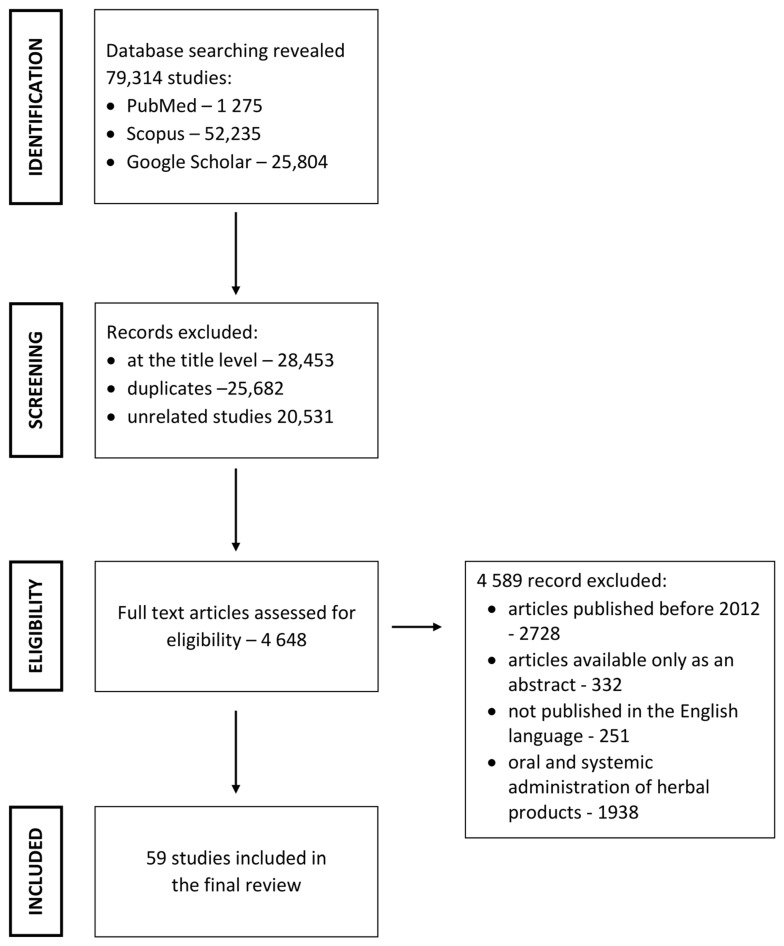
Search strategy used to identify relevant articles.

Overall, 79,314 articles were found in the databases. Of these, 74,666 articles were excluded at the title level; among them were also duplicates and unrelated articles. Furthermore, 4589 articles were excluded as not meeting the inclusion criteria. Finally, 59 articles were used for the review (Figure 1).

## 3. Herbal Products and Their Active Constituents Used for Diabetic Wound Healing

### 3.1. Animal-Based Studies

Animal-based studies are important in the research on the use of herbal products and their active constituents for diabetic wound healing (Table 1, Table 2 and Table 3). The most described animal studies are based on diabetes induced by streptozotocin (STZ) and alloxan monohydrate. Alloxan monohydrate and STZ are the most popular diabetogenic agents used for assessing the anti-diabetic or hypoglycemic capacity of test compounds. These compounds are cytotoxic glucose analogs that preferentially accumulate in pancreatic β cells via the GLUT2 glucose transporter [12]. STZ is a glucosamine–nitrosourea compound, a cytostatic antibiotic produced by *Streptomyces achromogenes,* used clinically as a chemotherapeutic agent in the treatment of pancreatic β-cell carcinoma. STZ damages pancreatic β cells, resulting in hypoinsulinemia and hyperglycemia [13]. Moreover, STZ induces type 1 and 2 diabetes in rodents [14]. Alloxan monohydrate, a urea derivative, selectively inhibits glucose-induced insulin secretion through its ability to inhibit the β cell glucose sensor glucokinase [12]. Inhibition of glucokinase reduces glucose oxidation and ATP generation, thereby suppressing the ATP signal that triggers insulin secretion [12]. Alloxan monohydrate can induce type 1 diabetes [15]. In a few reported studies, induction of diabetes was based on the combination of STZ with a high-fat diet (STZ/HFD) [16] or carried out on genetically modified animals with diabetes (leptin receptor-deficient (Leprdb/JNju, db/db) mice) [17]. Due to single-gene mutations that lead to the lack of action by the satiety factor leptin or its cognate receptor, these rodents spontaneously develop severe hyperphagia leading to obesity and manifest some type 2 diabetes mellitus (T2DM)-like characteristics [18].

The wound healing activity of herbal products and their active constituents conducted on animals with induced diabetes are mainly based on the excision wound model and one study on the punch wound model [19]. The excision wound model is induced by the removal of some part of the skin at the depth of the epidermis and upper dermis (a partial thickness (or split-thickness) wound) or both epidermis and dermis up to the fascia or subcutaneous tissue (a full-thickness wound) [20]. Excision wounds well illustrate the skin defects that can be observed in diabetic wounds and allow the evaluation of re-epithelialization and wound closure. All herbal products and their active constituents (Table 1, Table 2 and Table 3) showed wound contraction in a shorter time, increased wound breaking strength, increased re-epithelialization, and better granulation compared to that of standard drugs (5% and 10% povidone-iodine, 1% silver nitrate, 1% silver sulfadiazine, 2% mupirocin, 8.5% mafenide, bacitracin) and commercially available wound dressings (Comfeel—hydrocolloid dressing, Kaltostat—alginate dressing) as positive controls.

### 3.2. Human-Based Studies

In comparison to many animal-based studies, there are only a few clinical trials describing the influence of herbal products on diabetic wound healing (Table 4). Unfortunately, there are no human-based studies showing the effect of active compounds isolated from herbal products on diabetic wound healing. This imbalance between animal-based studies and human-based studies arises from the fact that clinical trials designed to prove herbal products efficacy and safety are expensive, long-lasting, and require unique regulatory pathways and special permission from regulatory bodies. Despite these limitations, some scientific studies are being performed.

**Table 1:** Herbal products used for diabetic wound healing, animal-based studies.

Herbs	Model of the Study	Pharmacological Data	Effect	Mechanism of Action	Antimicrobial Activity	Ref.
*Aloe vera*	STZ-induced diabetic Wistar rat, excision wound model	*A. vera* gel; control 1: untreated group; control 2: untreated diabetic group; treatment: once a day for 9 days	significantly increases level of GAGs and breaking strength on day 9	-	-	[21]
*Aloe Vera, Adiantum capillus veneris, Commiphora molmol, henna*	STZ- induced diabetic Wistar rats; excision wound model	ointment with herbal powder mixed in equal parts with Vaseline; control 1: untreated non-diabetic group; control 2: untreated diabetic group; control 3: Vaseline; treatment: once a day for 21 days	better wound closure; expression of the Mmp9 gene decreased significantly in diabetic group after 14 days	-	-	[22]
*Aloe vera,* *Nigella sativa*	AM-induced diabetic Wistar rats; excision wound model	*N. sativa* oil gel (NSO); *Aloe vera* gel (AV); control: untreated group; treatment: 100 mL of gel and transparent film dressing	significantly smaller wound area in AV than NSO group; necrotic tissue and inflammation decreased in AV group compared with NSO group; re-epithelialization was better in AV than NSO group	-	-	[23]
*Aloe vera,* *Teucrium polium*	STZ-induced diabetic BALB/c mice; excision wound model	5% and 10% *T. polium* hydroethanolic extract in ointment; 5% and 10% *A. vera* gel in ointment; combination of 5% *T. polium* extract and 5% *A. vera* gel in ointment; positive control: mupirocin; treatment: once a daily for 14 days	mixed herbal ointment shortened the inflammatory phase and reduced the levels of tissue MDA, TNF-α, and IL-1β compared to mupirocin; fibroblast proliferation, collagen deposition, and expression of VEGF, IGF-1, GLUT-1, and FGF-2 were significantly increased by all herbal ointments	anti-inflammatory activity	-	[24]
*Agrimonia pilosa;* *Nelumbo nucifera; Boswellia carteri; Pollen typhae*	STZ-induced diabetic C57BL/6 mice; excision wound model	mixed powder of *A. pilosa*, *N. nucifera*, *B. carteri*, *P. typhae* (ANBP); control: untreated group; treatment: once a day for 21 days	accelerated wound healing, promoted vascularization, and inhibited inflammation	angiogenic activity; anti-inflammatory activity	-	[25]
*Annonas quamosa*	STZ-induced diabetic Wistar rats; excision wound model	*A. squamosa* ethanolic extract; control 1: untreated non-diabetic group; control 2: untreated diabetic group; treatment: 200 μL, once daily	better wound healing through increased levels of enzymatic and non-enzymatic antioxidants in wound tissues	antioxidant activity	-	[26]
*Arnebia euchroma, Pistacia atlantica*	AM-induced diabetic Wistar rats; excision wound model	10% *A. euchroma* extract in Eucerin; 5% *A. euchroma* extract and 5% *P. atlantica* EO in natural cow oil; 10% *A. euchroma* extract and 10% *P. atlantica* EO in natural cow oil; 10% *A. euchroma* extract and 10% *P. atlantica* EO in Eucerin; positive control: honey; negative control: Eucerin; treatment: once a day	the most effective in wound healing was 5% *A. euochroma* and gum mixture of animal oils	-	-	[27]
*Azadirachta indica, Glycyrrhiza glabra, Ficus infectoria, Shorea robusta, Curcuma longa, Berberis aristata, Rubia cordifolia, Pongamia glabra, Ficus religiosa, Ficus bengalensis, Centella asiatica*	AM-induced diabetic Wistar rats; incision and excision wound models	Cream A (extracts of *G. glabra*, *F. infectoria*, *S. robusta*, *C. longa*, *B. aristata*, *R. cordifolia*, *A. indica*, *P. glabra*, Yashad Bhasma as Ayurvedic preparation); Cream B (extracts of *F. religiosa, F. bengalensis*, *C. asiatica*, *S. robusta, G. glabra*, *A. indica*, *P. glabra*, Jatyadi Oil, and Yashad Bhasma); positive control: framycetin sulfate cream; treatment: once a day for 10 days (incision method) and 16 days (excision method)	Cream B was found to be more an effective wound healing agent than cream A and framycetin	-	-	[28]
*Blepharis* *maderaspatensis*	STZ-induced diabetic Wistar rats; excision wound model	paste formula of 10 g, 15 g, 20 g of extract, 60 g black powder mixed with egg white and 2 or 3 drops of lime juice q.s.; negative control: untreated group; positive control: 1% framycetin sulphate; treatment: twice every day until the wound healed completely	paste with 20% extract completely healed wounds by 18th day of treatment	-	-	[29]
*Butea monosperma*	AM-induced diabetic Wistar rats; excision wound model	20% w/w methanolic flower extract in white petroleum jelly; control: Vaseline; positive control: soframycin ointment; treatment: 11 days	wound contraction	-	-	[30]
*Camellia sinensis*	AM-induced diabetic Wistar rats; incision and excision wound models	0.6% green tea methanolic extract; control 1: nontreated diabetic group; control 2: untreated non-diabetic group; control 3: Vaseline; positive control: 5% w/w povidone iodine; treatment: twice daily until completely healed	faster wound contraction; increased collagen and fibronectin deposition with higher expression of NO; promoted angiogenesis process via molecular control of circulating hypoxia-responsive microRNAs: miR-424, miR-210, miR-199a, and miR-21	angiogenic activity	-	[31]
*Cassia auriculata*, *Mangifera indica*, *Ficus banghalensis*, *Cinnamomum tamala*, *Trichosynthis diocia*	STZ-induced diabetic Wistar rats; incision, excision, dead space models	aqueous extracts mixed in equal proportions in polyherbal formulation; control 1 and 2: untreated non-diabetic and diabetic groups; positive control: 5% glibenclamide ointment; treatment: once a day for 18 days (excision model) or until wound was healed (incision model)	significant increase in wound breaking strength, epithelialization, and level of hydroxyproline	antioxidant activity	-	[32]
*Cotinus coggygria*	STZ-induced diabetic Wistar rats; excision wound model	5% (*w*/*w*) ethanol extract of *C. coggygria* ointment; control: untreated group; treatment: 0.5–1 g, once a day for 14 days	significantly increased hydroxyproline content and elevation in GSH, statistically significant decrease in MDA level in the treated group vs. control group	antioxidant activity, anti-inflammatory activity	-	[33]
*Cymbopogon nardus*	STZ-induced diabetic Swiss albino mice; excision wound model	*C. nardus* EO dispersed in 100 mL of olive oil; control 1: saline-treated diabetic group; control 2: *C. albicans*-infected diabetic group; positive control: clotrimazole (1 mg/day) dispersed in 100 mL of olive oil; treatment: 25 mg once a day for 21 days	attenuated the growth of the fungus on diabetic wounds and simultaneously reduced the inflammation which leads to acceleration of the wound healing process	anti-inflammatory activity, antifungal activity	*C. albicans,* *C. glabrata,* *C. tropicalis*	[34]
*Euphorbia hirta*	AM-induced diabetic Swiss albino rats; excision wound model	5% and 10% ethanolic extract of *E. hirta* ointment; positive control: 5% povidone iodine ointment; treatment: once a day for 16 days	significant wound closure	-	-	[35]
*Hypericum* *perforatum*	STZ-diabetic Sprague–Dawley rats; incision and excision wound model	*H. perforatum* in olive oil; control 1: untreated non-diabetic group; control 2: untreated diabetic group; control 3: olive oil; treatment: once a day for 21 days	faster inflammatory response and better healing; significantly higher tensile strength, tissue hydroxyproline concentration, and collagen density	anti-inflammatory activity	-	[36]
*Hypericum* *perforatum*	STZ-induced diabetic Wistar rats; excision wound model	5% and 10% *H. perforatum* gel; control 1: untreated group; control 2: gel base; treatment: once a day for 15 days	faster wound closure rate, improved tissue regeneration by enhancing fibroblast proliferation, collagen bundle synthesis, and revascularization	angiogenic activity	-	[37]
*Lantana camara*	AM-induced diabetic rats; excision wound model	10% ethanolic extract of *L. camara* emulgel; control 1: untreated non-diabetic group; control 2: untreated diabetic group; positive control: soframycin ointment; treatment: twice daily for 12 days	faster wound closure and reduced epithelization period	-	-	[38]
*Lycium depressum*	STZ-induced diabetic Wistar rats; incision and excision wound model	1 g, 2 g, 4 g powder of methanolic *L. depressum* extracts in ointment; control 1: untreated group; control 2: base formulation; treatment: once a day	enhanced wound contraction, decreased epithelialization time, increased hydroxyproline content	-	-	[39]
*Momordica* *charantia*	STZ-induced diabetic Sprague–Dawley rats;excision wound model	*M. charantia* fruit extract powder and ointment; control 1 and 2: untreated non-diabetic and diabetic group; control 3: ointment base; positive control: povidone iodine ointment; treatment: once a day for 10 days	faster wound closure rate; intense TGF-β expression	angiogenic activity	-	[40]
*Moringa oleifera*	STZ-induced diabetic Wistar rats; excision wound model	0.5%, 1%, and 2% *w*/*w* aqueous fraction of *M. oleifera;* control 1: non-diabetic group; control 2: diabetic group; positive control: 1% *w*/*w* silver sulfadiazine; treatment: once a day for 21 days	decreased wound size, improved wound contraction, tissue regeneration; downregulation of inflammatory mediators, such as TNF-α, IL-1β, IL-6, iNOS synthase, COX-2, and upregulation of VEGF	angiogenic activity; anti-inflammatory activity	*S. aureus*, *P. aeruginosa*, *E. coli*	[41]
*Nigella sativa*	STZ-induced diabetic Wistar rats; excision wound model	20% and 40% hydroethanolic *N. sativa* extracts ointment; control 1: untreated non-diabetic group; control 2: Eucerin-treated non-diabetic group; control 3: phenytoin (1%)-treated non-diabetic group; control 4: untreated diabetic group; control 5: phenytoin (1%)-treated diabetic group; treatment: 21 days	the shortest duration of wound healing in diabetic *N. sativa* extract (40%)-treated group (15 days) followed by diabetic *N. sativa* (20%)-treated group (18 days)	anti-inflammatory activity	*-*	[42]
*Pelargonium* *graveolens, Olive* *riadecombens*	STZ-induced diabetic Wistar rats; excision wound model	formulations with 1% EOs alone; mixture with 1% *P. graveolens* and 1% *O. decombens*; control 1: basic formula; control 2: saline; treatment: once a day for 30 days	reduction of wound size; highest tissue repair in EO mixture group	-	-	[43]
*Piper betel*	STZ-induced diabetic Sprague-Dawley rats; excision wound model	paste with powder of *P. betel* and 0.9% saline; control 1: untreated non-diabetic rats; control 2: untreated diabetic rats; positive control: 1% silver nitrate cream; treatment: once a day for 7 days	significant increase in hydroxyproline content and SOD; decreased MDA level; decrease in 11b-HSD-1 expressions	antioxidant activity	-	[44]
*Plantago lanceolata, Arnica montana, Tagetes patula*, *Symphytum* *officinale, Calendula officinalis*, *Geum urbanum*	STZ-induced diabetic Wistar rats; excision wound model	mixture of alcoholic herbal extract-loaded chitosan formulation; control 1: chitosan formulation; positive control: Betadine ointment; treatment: once daily for 14 days	wound contraction and accelerated wound healing process; more complete re-epithelialization and denser collagen deposition	antioxidant activity	-	[45]
*Prosopis farcta*	STZ-induced diabetic Wistar rats; excision wound model	fruit powder and root extract of *P. farcta*; control 1: untreated non-diabetic group; control 2: untreated diabetic group; treatment: twice a day for 15 days	fruit powder and root extract accelerated wound healing	-	-	[46]
*Psidium guajava*	AM-induced diabetic Wistar rats; excision wound model	gel with 5% and 10% (*w*/*w*) of tannin-enriched fraction of *P. guajava* leaves; control 1: saline; control 2: gel without tannin fraction; positive control: *Aloe vera* gel 90% *w*/*w*; treatment: daily for 12 days	wound contraction	-	-	[47]
*Salvia kronenburgii; Salvia euphratica*	STZ-induced diabetic Wistar rats; incision and excision wound models	0.5% and 1% (*w*/*w*) ethanol extracts ointment; control 1: untreated group; control 2: ointment base; positive control: Fitocream with 15% (*w*/*w*) *Triticum vulgare* L. aqueous extract; treatment: 0.5 g ointments, topically once daily for 14 days	wound contraction; increased re-epithelialization and angiogenesis, decreased dermal inflammation; oxidative damage to DNA was reduced on day 7 for *S. euphratica* ointment and on day 14 for *S. kronenburgii* ointment	angiogenic activity; antioxidant activity	*S. aureus*, *E. coli*, *A. baumannii*, *A. hydrophila*, *M. tuberculosis*, *C. glabrata*, *C. parapsilosis*, *C. tropicalis*	[48]
*Solanum* *xanthocarpum*	STZ-induced diabetic Wistar rats; inclusion and exclusion wound model	5% and 10% extract of *S. xanthocarpum*gel; control 1: non-diabetic group with gel base; control 2: diabetic group with gel base; positive control: *A. vera* cream and juice; treatment: once a day for 14 days	significant increase in collagen, hexosamine, hyaluronic acid, lipid peroxidation, NO; reduced levels of pro-inflammatory cytokines (IL-1β, IL-6, and TNF-α); enhanced level of VEGF	anti-inflammatory activity	-	[49]
*Stryphnodendronadstringens*	STZ-induced diabetic Wistar rat, excision wound model	1% crude extract gel; control 1: base gel; treatment: once a day for 14 days	stimulation of the production of collagen fibers at the wound site; increased upregulation of COX-2 and VEGF	anti-inflammatory activity	-	[50]
*Quercus infectoria*	STZ-induced diabetic Wistar rats, excision wound model	30% *w/v Q. infectoria* formulation; control: saline; treatment: 15 mL once a day until wound closure	enhanced the wound healing process with abundant cellular infiltration, collagen deposition, and re-epithelialization	antioxidant activity; antimicrobial activity	MRSA	[51]

Legends: 11b-HSD-1—11β-Hydroxysteroid dehydrogenase type 1; AM—alloxan monohydrate; COX-2—cyclooxygenase-2; EO—essential oil; FGF-2—fibroblast growth factor-2; GAG—glycosaminoglycan; GLUT-1—glucose transporter-1; GSH—glutathione, IGF-1—insulin-like growth factor 1; IL-1β—interleukin-1β; IL-6—interleukin 6; iNOS—inducible nitric oxide synthase; MDA—malondialdehyde; MRSA—methicillin-resistant *Staphylococcus aureus*; NO—nitric oxide; SOD—superoxide dismutase; STZ—streptozotocin; TGF-β—transforming growth factor-β; TNF-α—tumor necrosis factor-α; VEGF—vascular endothelial growth factor.

**Table 2:** Active constituents isolated from herbal products used for diabetic wound healing, animal-based studies.

Herbal Products	Model of the Study	Pharmacological Data	Effect	Mechanism of Action	Ref.
20(S)-protopanaxadiol from *Panax* *notoginseng*	leptin receptor-deficient (Leprdb/JNju, db/db) mice; excision wound model	20(S)-protopanaxadiol (PPD); control: PBS; treatment: 15 μL of PPD (0.6, 6, and 60 mg/mL) or PBS every other day for 14 days	PPD accelerated wound closure and epithelial gaps, elevated VEGF expression and capillary formation; PPD stimulated angiogenesis via HIF-1α-mediated VEGF expression by activating p70S6K through PI3K/Akt/mTOR and Raf/MEK/ERK signaling cascades	angiogenic activity	[17]
arnebin-1 from *Arnebia euchroma* (Zicao)	AM-induced diabetic Sprague–Dawley rats; punch wound model	0.1% arnebin-1 ointment; control 1: non-diabetic untreated group; control 2: untreated diabetic group; control 3: diabetic group with vehicle ointment; treatment: once a day for 7 days	significantly increased wound closure rate; reduced number of macrophages, increased number of fibroblasts, remarkable degree of neovascularization and epithelization; synergetic effect with VEGF	angiogenic activity	[19]
kaempferol	STZ-induced diabetic Wistar rats; incision and excision wound model	0.5% and 1% (*w*/*w*) kaempferol (KM) ointments; control 1: untreated non-diabetic group; control 2: untreated diabetic group; control 3: ointment base; treatment: 0.5 g ointment, once a day for 14 days	the best wound healing effect using 1% KM ointment; increased hydroxyproline and collagen; improved wound resistance (tensile strength), wound closure, and accelerated re-epithelialization	antioxidant activity; anti-inflammatory activity	[52]
kirenol from *Siegesbeckia orientalis*	STZ-induced diabetic Wistar rats; excision wound model	diabetic and non-diabetic rats treated with 15% and 30% kirenol; treatment: once a day for 14 days	wound closure, enhanced granule-forming tissue with noticeable propagation of fibroblasts, amplified vascular initiation, and sediment of collagen fibers; decreased NF-κB, COX-2, iNOS, MMP-2, and MMP-9 levels	angiogenic activity; anti-inflammatory activity	[53]
luteolin	STZ-induced diabetic Wistar rats; incision and excision wound models	0.5% and 1% (*w*/*w*) luteolin ointments; control 1: untreated non-diabetic group; control 2; untreated diabetic group; control 2: ointment base; treatment: once daily for 14 days	the best wound healing activity was observed in incision and excision wounds treated with 0.5% (*w*/*w*) luteolin ointment	-	[54]
luteolin; flavonoids fraction from *Martynia annua*	STZ-induced diabeticWistar rats; excision wound model	0.2% and 0.5% *w*/*w* of luteolin and flavonoid fraction ointment; control 1: untreated group; control 2: ointment base; positive control: 5% povidone iodine; treatment: twice daily	enhanced wound healing through free radical-scavenging activity	antioxidant activity	[55]
neferine from *Nelumbo nucifera* (lotus)	STZ-induced diabetic Wistar rats; excision wound model	10% neferine; control 1: untreated non-diabetic group; control 2: untreated diabetic group; control 3: untreated diabetic group with excision wound treatment: once a day for 14 days	significant wound closure rate, decrease in the period of re-epithelialization, higher amount of collagen and protein content; mRNA level of Nrf-2, collagen-1, TGF-β, and α-SMA were decreased, and Kaep-1 was significantly increased; downregulation of inflammatory mediators (NF-κβ, TNF-α, IL-1β, IL-8, iNOS, and COX-2) and upregulation of GFs	anti-inflammatory activity	[56]
pongamol; flavonoid-rich fraction from *Tephrosia purpurea*	STZ-induced diabetic rats, excision wound model	ointments with 5% (*w*/*w*) flavonoid-rich fraction and 0.2 and 0.5% (*w*/*w*) pongamol (PONG); positive control: povidone iodine; treatment: once a day for 20 days	100% wound contraction; increased hydroxyproline and enzyme levels (SOD, CAT, and GSH), matured collagen fibers and fibroblasts with better angiogenesis	antioxidant activity, angiogenic activity	[57]
*quercetin*	STZ- induced diabetic Wistar rats; excision wound model	quercetin ointment (1 g quercetin mixed with 99 g of petroleum jelly); control: petroleum jelly; positive control: 5% povidone ointment; treatment: once a day for 21 days	increased wound healing	-	[58]

Legends: α-SMA—Smooth muscle alpha-actin; AM—alloxan monohydrate; CAT—catalase; COX-2—cyclooxygenase-2; GF—growth factor; GSH—glutathione; HIF-1α—hypoxia-inducible factor 1α; IL-1β—interleukin-1β; iNOS—inducible nitric oxide synthase; Kaep-1—Kelch-like ECH-associated protein *1;* MMP—matrix metalloproteinase; NF-κB—nuclear factor kappa-light-chain-enhancer of activated B cells; Nrf-2—nuclear factor erythroid 2-related factor 2; SOD—superoxide dismutase; STZ—streptozotocin; TNF-α—tumor necrosis factor-α; TGF-β—transforming growth factor-β; VEGF—vascular endothelial growth factor.

**Table 3:** Herbal products and their active constituents loaded on dressings used for diabetic wound healing, animal-based studies.

Herbs	Model of the Study	Pharmacological Data	Effect	Mechanism of Action	Antimicrobial Activity	Ref.
*Aloe vera,* *Hypericum perforatum*	STZ-induced diabetic Wistar rats; excision wound model	15% *A. vera* gel with poly ε-caprolactone/gelatin (PCL/Ge) in nanofiber dressings; 15% *H. perforatum* oil with PCL/Ge in nanofiber dressings; control 1: PCL/Ge; control 2: *A. vera*gel/*H. perforatum* oil; positive control: 10% povidone-iodine; treatment: wound was covered with dressings after 7th day of STZ induction	*H. perforatum* oil gel-based nanofibers was better than *A. vera* gel-based nanofibers for wound healing	-	-	[59]
apigenin from *Morus alba*	STZ-induced diabetic Wistar rats; excision wound model	apigenin (APN)-loaded hydrogels (HGs) with gellan gum–chitosan (GGCH) and PEG as a cross-linker (APN-loaded GGCH-HGs); control 1: vehicle (GGCH-HGs); positive control: Betadine; treatment: 18 days	APN GGCH-HGs effectively stimulated wound contraction with significant antioxidant activity and increased collagen content; increased level of SOD and CAT in granuloma tissue of APN-treated group	antioxidant activity	-	[60]
*Blechnum orientale*	STZ-induced diabetic rats; excision wound model	hydrogel (sodium carboxymethyl-cellulose) with 4% *w*/*w B. orientale* extract; treatment: once a day for 14 days	wound closure at 12 days; re-epithelialization, higher fibroblast proliferation, collagen synthesis, and angiogenesis	antioxidant activity; antibacterial activity; angiogenic activity	MRSA	[61]
curcumin	STZ-induced diabetic Wistar rats; excision wound model	nanohybrid scaffold incorporating curcumin-loaded chitosan nanoparticles (CUR-CSNPs) impregnated into collagen–alginate (COL/ALG); control 1: sterile gauze; control 2: COL/ALG scaffold without CUR-CSNPs; treatment: once a day for 15 days	faster wound closure, complete epithelialization with thick granulation tissue formation; lack of compact collagen deposition in placebo scaffold group; presence of inflammatory cells in control group	-	-	[62]
curcumin from *Curcuma longa*	STZ-induced diabetic Sprague– Dawley rats; excision wound model	curcumin-loaded gum tragacanth/poly(ε-caprolactone) electrospun nanofibers (GT/PCL/Cur nanofibers); control: untreated diabetic group; treatment: wounds were wrapped with GT/PCL/Cur nanofibers for 15 days	wound closure with well-formed granulation tissue dominated by fibroblast proliferation, collagen deposition, complete early regeneration of epithelial layer; formation of sweat glands and hair follicle tissue; increased amount of angiogenesis, granulation tissue area, and fibroblast numbers, and decreased epithelial gap	angiogenic activity; antibacterial activity	MRSA, ESBL Gram-negative bacteria	[63]
curcumin, *Lithospermi radix*	STZ-induced diabetic Sprague–Dawley rats; excision wound model	1 μg/mL curcumin and 625 μg/mL *L. radix* extract loaded in GC/L/C bilayer nanofibrous scaffolds (gelatin/PVA solution with curcumin and extract was electrospun onto the chitosan scaffolds); control 1: gauze; GC membrane, GC/L membrane, GC/C membrane, GC/L/C scaffold; positive control: Comfeel^®^; treatment: once a day for 14 days	decreased levels of pro-inflammatory markers (IL-6, TNF-α) provided evidence for the anti-inflammatory effects of GC/L/C treatment; increase in recovery rate of wound on day 7	anti-inflammatory activity	-	[64]
hydroxysafflor yellow A from *Carthamus* *tinctorius*	STZ-induced diabetic Sprague–Dawley rats; excision wound model	hydroxysafflor yellow A and deferoxamine (HSYA/DFO) loaded in chitosan/gelatin hydrogels in ratio of 5:5; control 1: PBS; control 2: hydrogel base; control 3: HSYA and DFO solution; treatment: once a day for solutions/once every 2 days for hydrogel for 16 days	HSYA/DFO exerted synergistic effect on enhancing angiogenesis by up regulation of HIF-1α expression	angiogenic activity	-	[65]
*Malva sylvestris*	STZ-induced diabetic Wistar rats; excision wound model	nanofibers of polyurethane and carboxymethyl cellulose (PU/CMC) with 15% *w*/*w M. sylvestris* extract; control group: gauze bandage and PU/CMC; treatment: once a day for 14 days	higher collagen deposition and neovascularization; increased macrophage infiltration and fibroblast proliferation on day 7; enhanced collagenization and epithelium regeneration on day 14	anti-inflammatory activity, angiogenic activity	*S. aureus;* *E. coli*	[66]
*Moringa oleifera*	STZ/HFD-induced diabetic Sprague–Dawley rats; excision wound model	0.1, 0.5, and 1% *M. oleifera* leaves (MOL) aqueous extract loaded in hydrocolloid film dressing; control 1: untreated non-diabetic group; control 2: untreated diabetic group; positive control: Kaltostat; treatment: once a day for 21 days	0.5% film significantly enhanced the wound closure at day 7; high collagen deposition and complete re-epithelialization after treatment with 0.5 and 1% MOL hydrocolloid film dressing	-	-	[16]
polysaccharide from *Astragali Radix*	STZ-induced diabetic Sprague–Dawley rats; excision wound model	*Astragalus* polysaccharide (APS)-loaded tissue engineering scaffolds (TES); control 1: untreated healthy group; control 2: TES alone; treatment: 5 mg each, once a day for 12 days	APS in TES mimics structure of extracellular matrices and restored skin microcirculation; faster collagen synthesis, wound closure, and appendage and epidermal differentiation	angiogenic activity	-	[67]
polysaccharide from *Curcuma zedoaria*	STZ-induced diabetic Sprague–Dawley rats; excision wound model	polysaccharide (ZWP) in chitosan/silk hydrogel sponge loaded with platelet-rich plasma (PRP) exosomes (PRP-Exos/ZWP); control 1: gauze containing 100 μL PBS; control 2: chitosan/silk hydrogel group; control 3: chitosan/silk hydrogel sponge loaded with PRP exosomes (PRP-Exos); treatment: wound dressings changed every 3 days for 15 days	wound closure, up regulation of collagen synthesis and deposition, and angiogenesis at the wound site were observed for PRP-Exos/ZWP	angiogenic activity	-	[68]
polysaccharide from *Periplaneta americana*	STZ-induced diabetic Wistar rats; excision wound model	hydrogel (carbomer 940, carboxymethyl cellulose) with polysaccharide *P. americana*; control 1: saline-treated non-diabetic group; control 2: saline-treated diabetic group; control 3: hydrogel base; positive control: Kangfuxin solution; treatment: once daily for 15 days	polysaccharide hydrogel effectively accelerated wound healing; increased inflammation alleviation, angiogenesis, and macrophage polarization	anti-inflammatory activity; angiogenic activity	-	[69]
resveratrol	STZ-induced diabetic Wistar rats; excision wound model	resveratrol solution; resveratrol-loaded microparticles; resveratrol loaded microparticle impregnated dermal matrix (DM-MP-RSV) control 1: untreated non-diabetic group; control 2: untreated diabetic group; control 3: untreated diabetic wound group; control 4: dermal matrix (DM); treatment: once a day for 14 days	the highest healing score in the DM-MP-RSV group with an increased antioxidant activity	antioxidant activity	-	[70]
vicenin-2	STZ-induced diabetic Sprague–Dawley rats; punch wound model	12.5, 25, and 50 μM Vicenin-2 hydrocolloid film (sodium alginate); control 1: non-diabetic, blank film-treated; control 2: diabetic; blank film-treated; positive control: 316 μM allantoin film; treatment: 0.8 cm^2^ film dressing and adhesive-permeable bandage wrapping; every day for 14 days	enhanced diabetic wound healing; reduced pro-inflammatory cytokines (IL-1β, IL-6, and TNF-α), mediators (iNOS and COX-2), and NO via the NF-κB pathway; enhanced cell proliferation, migration, and wound contraction via the VEGF and TGF-β pathways	anti-inflammatory activity	-	[71]

Legends: CAT—catalase; COX-2—cyclooxygenase; ESBL—extended spectrum beta-lactamase; HIF-1α—hypoxia-inducible factor 1α; IL-1β—interleukin 1β; IL-6—interleukin 6; iNOS—inducible nitric oxide synthase; MRSA—methicillin-resistant *Staphylococcus aureus*; NF-κB—nuclear factor kappa-light-chain-enhancer of activated B cells; NO—nitric oxide; SOD—superoxide dismutase; STZ—streptozotocin; TGF-β—transforming growth factor-β; TNF-α—tumor necrosis factor-α; VEGF—vascular endothelial growth factor.

**Table 4:** Herbal products used for diabetic wound healing, human-based studies.

Herbs	Model of the Study	Pharmacological Data	Effect	Ref.
*Actindia deliciosa* (kiwifruit)	randomized clinical trial; 37 patients with neuropathic diabetic foot ulcer; - 20 patients in control group; - 17 patients in experimental group	pure extract of kiwifruit; treatment: twice daily for 21 days	reduction in surface area of foot ulcer; significantly higher amounts of collagen and granulation tissues; significantly higher levels of angiogenesis; no significant antibacterial activity	[72]
*Aloe vera*	random clinical trial; 60 patients with type 2 diabetes: - 30 patients—positive control group - 30 patients with intervention	2% *A. vera* ointment; positive control: Betadine; treatment: once a day for 14 days	accelerated wound healing	[73]
*Centella asiatica,* *Plectranthus* *amboinicus*	single-center, randomized, controlled, open-label study; 24 diabetic foot ulcer patients: - 12 patients—WH-1 cream; - 12 patients—hydrocolloid fiber dressings	1.25% WH-1 cream (fraction of PA-F4 from *P. amboinicus* and S1 from *C. asiatica* in 1:4 ratio); treatment: twice daily for 14 days	no statistically significant differences in wound size after WH-1 cream application	[74]
olive oil	double-blind, randomized clinical trial; 34 patients with Wagner’s ulcer grade 1 or 2: - 17 patients—topical olive oil, oral antibiotics, local wound debridement, routine care; - 17 patients—oral antibiotics, local wound debridement, routine care	treatment: once a day for 4 weeks; routine care: ulcers cleaned with 1000 mL sterile 0.9% saline solution every day, after drying, wound was dressed with sterile gauze and latex-free tape	complete ulcer healing in olive oil group	[75]
*Securinega leucopyrus*	case study, one patient with chronic diabetic wound	paste of *S. leucopyrus*in sesame oil; treatment: once daily for 15 days	complete healing after one-month treatment	[76]

The human studies are based on the diabetic foot ulcer (DFU) model. The DFU model is closely associated with peripheral vascular disease, neuropathy, and the chronic non-healing nature of wounds [77]. High glucose levels subsequently destruct the nerve fibers [78] and induce capillary size reduction, accelerating atherosclerosis and vasoconstriction, which collectively leads to occlusive arterial disease and DFU formation [79]. Moreover, DFUs, especially when they become infected, are a leading cause of morbidity and may lead to severe consequences, such as amputation. Optimal treatment of these diabetic foot problems usually requires a multidisciplinary approach, typically including wound debridement, pressure off-loading, glycemic control, negative pressure therapy, and surgical interventions [80]. These procedures allow the cleaning of the wound bed of excess exudate and microorganisms, and at the same time provides optimal conditions for tissue regeneration [80]. Topical application of *Aloe vera* [73], olive oil [75], kiwifruit [72], and *Securinega leucopyrus* [76] showed a reduction in surface area or complete foot ulcer healing. In turn, topical application of a cream with the active fraction isolated from *Plectranthus amboinicus* and *Centella asiatica* showed no statistically significant differences in wound size compared to hydrocolloid fiber dressings as a control [74].

## 4. Herbal Products and Their Active Constituents Loaded in Dressings Used for Diabetic Wound Healing

There is quite a lot of research on medicinal plant-based dressings for wound healing applications [81,82,83]. Some of them refer to diabetic wounds healing, including gauze, foams, drug-impregnated dressings (iodine, silver, polysaccharides), natural polymer-based dressing (hydrocolloids and hydrogel-based alginate, chitosan, collagen, cellulose), synthetic polymer-based dressings (poly (lactide-co-glycolide), polyurethanes, polyetheneglycols), and electrospun scaffolds [84]. Unfortunately, some types of traditional dressings can protect the wounds from the external environment but do not respond well to the wound-healing process. The most popular dressings for diabetic wounds based on herbal products and their active constituents are hydrogels, hydrocolloids, foams, and different nanofiber-based scaffolds (Table 3).

Hydrogels consist of natural or synthetic polymers and up to 70% water. These structures maintain a moist wound environment, which significantly accelerates the regeneration of the epidermis, prevents the risk of necrotic tissue formation, stimulates the process of autolytic wound cleansing, and inhibits the development of pathogens, which reduces the risk of wound infection, allergic reactions, and pain in the wound [85]. The advantages of hydrogels translate into the popularity of their use in the treatment of diabetic wounds. It was shown that polysaccharides isolated from *Periplaneta americana* and loaded onto a hydrogel (carbomer 940, carboxymethyl cellulose) [69], *Blechnum orientale* extract in a hydrogel (sodium carboxymethyl-cellulose) [61], apigenin-loaded hydrogel (gellan gum–chitosan with polyethylene glycol as a cross-linker) [60], and hydroxysafflor yellow A and deferoxamine loaded into hydrogels (chitosan/gelatin) [65] effectively stimulated wound contraction.

Hydrocolloids can absorb minimal to moderate amounts of wound fluids, and they can prevent water, bacteria, and oxygen from entering into the wound, as well as reduce the pH of the wound, inhibiting bacteria growth [84]. Unfortunately, hydrocolloid dressings are not appropriate for deeper and infected wounds that need oxygen to increase the healing rate of the wound. *Moringa oleifera* aqueous leaf extract (0.5%)-loaded hydrocolloid film dressings had proven to be the most promising approach to accelerate the diabetic wound healing process in both full-thickness excisions and partial thickness abrasion wounds in the HFD/STZ-induced diabetic type 2 model with comparable activity to commercial Kaltostat dressings [16]. In addition, vicenin-2 hydrocolloid film (sodium alginate) enhanced diabetic wound healing through increased cell proliferation, migration, and wound contraction [71].

Foam dressings consist of a porous structure that is excellent for absorbing large amounts of exudates, providing occlusion, protecting against bacteria and other infectious agents, promoting autolysis debridement, permeability to gases and water vapors, and are easy to remove [86]. *Gastrodia elata* extract and tea tree EO loaded in foam dressing containing silk fibroin protein accelerated wound recovery and achieved full closure of the wound within 21 days [87]. Moreover, histological analysis of regenerated skin tissues indicated that foam dressings enhanced the generation of thicker, denser, and more abundant collagen fibers in the dermis layer in comparison with the positive and negative control groups.

Nanofiber-based scaffolds offer a large surface area-to-volume ratio to allow cell adhesion and increase their exudate-absorbing capacity, antibacterial properties, and encapsulation of drugs for the desired period which helps in achieving their controlled release [88]. This release-controlling property is not provided by any of the dressings mentioned above nor by existing novel drug delivery systems (e.g., liposomes, nanostructured lipid carriers, nanoparticles, and dendrimers) used for topical applications [84]. Recently, wound dressings based on electrospun nanofiber scaffolds have attracted researchers’ attention since they replicate the characteristics of skin, have a high surface area-to-volume ratio, and tunable porous structure for easy nutrient infiltration and gas exchange [89]. Moreover, bilayer nanofibrous scaffolds reduce the frequency of dressing changes and minimize patients’ discomfort. Curcumin-loaded poly (ε-caprolactone) nanofibers as diabetic wound dressing increased the rate of wound closure and sustained release of curcumin for 72 h [90]. In addition, curcumin loaded in chitosan nanoparticles impregnated into collagen–alginate scaffolds [62] and bilayer nanofibrous scaffolds containing curcumin and *Lithospermi radix* extract (gelatin/poly(vinyl alcohol) solution with curcumin electrospun onto chitosan scaffolds) [64] showed faster diabetic wound closure. Polyurethane-based nanofiber wound dressings containing *Malva sylvestris* extract improved diabetic wound healing better than gauze bandage-treated wounds [66]. *H. perforatum* oil gel-based electrospun nanofibers showed better wound healing activity than *Aloe vera* gel-based electrospun nanofibers [59]. It was also shown that *Astragalus* polysaccharide-loaded tissue engineering scaffolds mimicked the structure of extracellular matrices and restored skin microcirculation, and increased collagen synthesis, wound closure, and appendage and epidermal differentiation [67]. The use of the antimicrobial activities and wound healing properties of herbal products and their active constituents loaded in dressing is a promising approach in diabetic wound healing and requires further research.

## 5. Herbal Products and Their Active Constituents Used for Diabetic Wound Infections

Prolonged diabetic wound infections are a factor in the delayed wound healing process. Moreover, if infected diabetic wounds are not treated properly, they could lead to systemic infection, sepsis, and even death. Furthermore, diabetic foot infections are the main cause of leg amputation. Diabetic wounds are more prone to microbial infections than normal wounds due to the high levels of blood glucose in the wound fluids that allow microbes to grow rapidly [91]. It was shown that an infected diabetic wound is more difficult and longer to heal compared to an uninfected diabetic wound. Kandimalla et al. [34] observed that fungal-infected wounds were not healed for up to 21 days whereas in non-infected diabetic wounds were healed by this period. Histopathology of the wound showed a wide area of necrosis with no signs of wound healing in infected diabetic wounds compared to normal diabetic wounds. Therefore, it is very important to implement a strict program for the prevention and treatment of diabetic foot ulcers, as well as proper management of microbial infections.

The most common bacteria detected in DFUs are superficial Gram-negative bacteria (*P. aeruginosa*, β-hemolytic *Streptococcus*, *Proteus* spp.) and Gram-positive bacteria (methicillin-susceptible *S. aureus*, methicillin-resistant *S. aureus*, β-hemolytic *Streptococcus*), and deeply penetrating anaerobes (*Peptostreptococcus* spp., *Bacteroides* spp., *Prevotella* spp., *Clostridium* spp.) [92,93,94,95]. Moreover, diabetic foot infections caused by bacteria such as *P. aeruginosa*, *Escherichia coli*, *Citrobacter* spp., *Acinetobacter* spp., and *Staphylococcus aureus* can develop into non-healing chronic wounds. Furthermore, the antibiotic susceptibility assay data of DFU isolates have also confirmed the distribution of multiantibiotic-resistant bacteria in the wound site of diabetic patients [96]. It was shown that all the Gram-positive isolates displayed resistance against penicillin and vancomycin, whereas *P. aeruginosa* had increased resistance against the most efficient antimicrobials such as ciprofloxacin (77%) and gentamycin (69%) [97].

Topical antimicrobial therapy is one of the most important methods of diabetic wound care. Herbal products and their active constituents are known to possess antimicrobial activity, even against resistant strains, making them a reliable source to combat diabetic wound infections [8,98]. Some researchers found that herbal products and their active constituents have strong antimicrobial activity against microorganism isolated from diabetic wounds or applied on infected diabetic wounds. It was found that quercetin and its esterified complex with 4-formyl phenyl boronic acid (4FPBA−Q) showed a remarkable effect against bacterial suspensions (1 × 10^5^ CFU/mL) containing Gram-positive (*S. aureus*) and Gram-negative (*P. aeruginosa, S. typhi*) bacteria isolated from diabetic foot ulcers [99]. *Malva sylvestris* extract loaded in polyurethane/carboxymethylcellulose nanofibers showed antibacterial against *S. aureus* and *E. coli* [66]. The aqueous fraction of *Moringa oleifera* was found to be active against *S. aureus*, *P. aeruginosa*, and *E. coli* [41]. Hydrogels with water extracts of Blechnum orientale [61] and *Quercus infectoria* [100] was active against the MRSA strain. Curcumin-loaded electrospun nanofibers showed antibacterial activity against MRSA and ESBL Gram-negative bacteria [63]. An herbal ointment with ethanol extracts of *Salvia kronenburgii* and *Salvia euphratica* showed antibacterial activity against *S. aureus*, *E. coli*, *A. baumannii*, *A. hydrophila*, and *M. tuberculosis,* as well as antifungal activity against *C. glabrata*, *C. parapsilosis*, and *C. tropicalis* [48]. Besides bacterial infections, diabetic wounds are complicated by fungal infections. *Candida* species are the most common yeast that infects diabetic wounds which leads to delays in the wound healing process [101]. *Cymbopogon nardus* EO dispersed in olive oil attenuated the growth of fungi (*C. albicans*, *C. glabrata*, *C. tropicalis*) on chronic diabetic wounds and simultaneously reduced the inflammation which led to acceleration of the wound healing process [34].

## 6. Mechanism of Action of Herbal Products and Their Active Constituents Used for Diabetic Wound Healing

**Figure 2:**
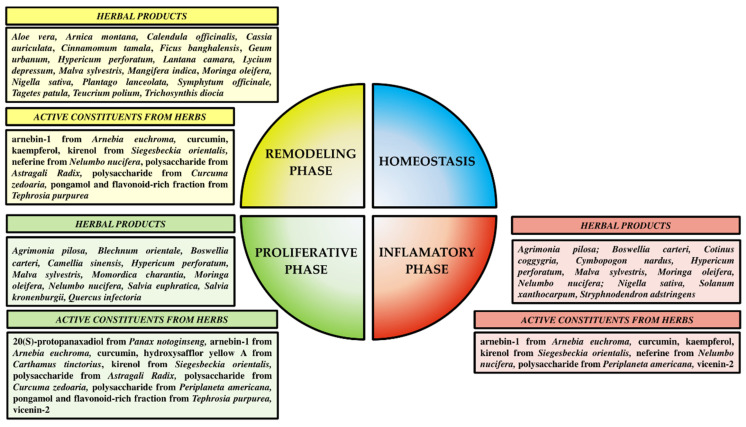
Influence of herbal products and their active constituents on the phases of diabetic wound healing.

Wound healing involves a complex sequence of events involving cellular and biochemical processes including four overlapping phases; (1) homeostasis (coagulation which controls excessive blood loss from the damaged vessels) (few minutes); (2) inflammatory (influx of macrophages and proteases to clean up debris and pathogens, secretion of growth factors and pro-inflammatory cytokines) (0–3 days); (3) proliferative (fibroblast migration, extracellular matrix formation, granulation, re-epithelialization, neoangiogenesis) (3–21 days), and (4) maturation or remodeling that occurs within the dermis (collagen crosslinking and reorganization, increase in the tensile strength of the extracellular matrix, scar formation) (21 day–2 years) [4,102]. These overlapping phases of wound healing as well as their length in diabetes patients are disturbed. Diabetic patients have a reduced ability to metabolize glucose resulting in hyperglycemic conditions which further complicate the wound healing process [103]. Hypoxia due to glycation of hemoglobin, leads to the alteration of red blood cell membranes and the narrowing of blood vessels, which further leads to a deficient supply of nutrients and oxygen to the tissue [104]. This local ischemia due to microvascular complications in diabetes considerably delays the wound healing processes. Serum glucose concentrations of more than 150 mL/dL were considered indicative of immune system dysfunction and leads to long-term inflammatory disease [105]. Microvascular complications, irregular inflammatory responses, impaired angiogenesis, tissue oxidative stress, impaired production of cytokines and growth factors, reduction of nitric oxide, impaired keratinocytes and fibroblast migration and proliferation, and abnormal levels of matrix metalloproteinases are the main factors that disturb the diabetic wound healing process [4]. Herbal products and their active constituents, through different mechanisms of action, affect the cellular and biochemical processes occurring in the different phases of wound healing (Figure 2).

### 6.1. Free Radicals and Oxidative Stress

Oxidative stress is caused by an increase in free radicals, reactive oxygen species (ROS), and/or reactive nitrogen species (RNS) in the body, which leads to intercellular biochemical dysregulation of the redox status [106]. The antioxidant system includes the major ROS-scavenging enzymes such as superoxide dismutase (SOD), catalase (CAT), and glutathione (GSH), and prevents damaging effects of oxidative stress [107]. An imbalance of free radicals and antioxidants in the body results in the overproduction of ROS which leads to cell/tissue damage, inflammation, neuropathy, ischemic lesion, and topical infection, delaying diabetic wound healing [108]. Therefore, decreasing ROS levels through antioxidative systems may improve diabetic wound healing.

Herbal products and their active constituents are well known for their antioxidant activity [109,110]. *Annona squamosal* ethanolic extract promoted increased levels of enzymatic and non-enzymatic antioxidants in wound tissues, thus detoxifying free radicals to promote better wound healing in normal and diabetic rats [26]. An ointment containing luteolin (0.5% *w*/*w*) and the flavonoid fraction (0.5% *w*/*w*) isolated from *Martynia annua* [55], a resveratrol solution, resveratrol-loaded microparticles, and resveratrol-loaded microparticles impregnated in a dermal matrix [70] enhancing diabetic wound healing through free radical scavenging. *Piper betel* paste significantly decreased the oxidative stress markers such as SOD and expression of 11β-*hydroxysteroid dehydrogenase* type 1 (11b-HSD-1) in diabetic wounds compared to untreated diabetic wounds [44]. Apigenin from *Morus alba* loaded into a hydrogel effectively stimulated diabetic wound contraction with significant antioxidant activity through increased levels of SOD and CAT in granuloma tissue [60]. Pongamol and flavonoid-rich fractions from *Tephrosia purpurea* ointment stimulated diabetic wound healing through antioxidant activity by increasing SOD, CAT, and GSH levels [57]. Hydrogels with 4% w/w *Blechnum orientale* extract exhibited stronger antioxidant activity compared to standards (ascorbic acid, α-tocopherol, BHT as butylohydroksytoluen, and Trolox-C as an analog of vitamin E) and effectively treated diabetic ulcer wounds [61]. An herbal formulation of *Cassia auriculata*, *Mangifera indica*, *Ficus banghalensis*, *Cinnamomum tamala*, and *Trichosynthis diocia*) [32], a mixture of alcoholic herbal extracts (*Plantago lanceolata, Arnica montana, Tagetes patula, Symphytum officinale, Calendula officinalis, Geum urbanum*) loaded onto chitosan [45], ethanol extracts of *Salvia kronenburgii* and *Salvia euphratica* ointment [48], *Cotinus coggygria* ointments [33], *Quercus infectoria* formulations [51], and kaempferol ointments [52] showed significant antioxidant activity and improved diabetic wound closure.

### 6.2. Impaired Inflammatory Cell Response

An increase in glucose levels and free fatty acids promotes the activation of macrophage-mediated inflammation in diabetes, contributing to the elevated production of pro-inflammatory cytokines [111]. M1-like macrophages with pro-inflammatory activity produce cytokines (IL-12, IL-1β, IL-6, TNFα, iNOS), while M2-like macrophages with anti-inflammatory activity are dominant in the proliferative phase of diabetic wound healing [112,113]. These recruit macrophages and immune cells that serve a pivotal role in orchestrating the appropriate healing of diabetic wound [114]. It was shown that macrophage dysregulation [115] and macrophage-derived IL-1β [116,117] lead to a prolonged inflammatory phase and impaired diabetic wound healing.

Herbal products and their active constituents have well-known anti-inflammatory activities which may be useful for the treatment of diabetic wound healing and its complications [118]. A combination of *Aloe vera* gel and *Teucrium polium* hydroethanolic extract in an ointment shortened the inflammatory phase and reduced the levels of tissue malondialdehyde (MDA), TNF-α, and IL-1β compared to the mupirocin positive control [24]. *Moringa oleifera* improved wound contraction through the downregulation of inflammatory mediators, such as TNF-α, IL-1β, IL-6, iNOS synthase, and COX-2 and the upregulation of VEGF [41]. *Solanum xanthocarpum* gel reduced levels of pro-inflammatory cytokines (IL-1β, IL-6, and TNF-α) and enhanced levels of VEGF [49]. Kirenol from *Siegesbeckia orientalis* affect wound closure by decreasing NF-κB, COX-2, iNOS, MMP-2, and MMP-9 levels [53]. Curcumin and *Lithospermi radix* extract loaded in bilayer nanofibrous scaffolds decreased levels of pro-inflammatory markers (IL-6 and TNF-α) and provided evidence for the anti-inflammatory effects of this treatment [64]. Neferine from *Nelumbo nucifera* (lotus) acts by down regulating inflammatory mediators (NF-κβ, TNF-α, IL-1β, IL-8, iNOS, and COX-2) and upregulating of GFs [56]. Vicenin-2 hydrocolloid films reduced levels of pro-inflammatory cytokines (IL-1β, IL-6, and TNF-α) and mediators (iNOS and COX-2), and NO via the NF-κB pathway [71]. It was also shown that macrophages/monocytes isolated from the wounds of diabetic mice treated with *Quercus infectoria* extract exhibited lower expression of the inflammatory cytokines IL-1β and TNF-α [100]. Polysaccharides from *Periplaneta americana* in a hydrogel [69] and *Malva sylvestris* extract in nanofibers [66] effectively accelerated wound healing through alleviation of inflammation and macrophage polarization. *Hypericum perforatum* oil extract [36], *Cotinus coggygria* ointment [33], hydroethanolic *Nigella sativa* extract ointment [42], *Cymbopogon nardus* essential oil dispersed in olive oil [34], *Salvia kronenburgii* and *Salvia euphratica* ointment [48], a mixed powder of *Agrimonia Pilosa*, *Nelumbo nucifera, Boswellia carteri*, and *Pollen typhae* [119], and a kaempferol ointment [52] reduced inflammation which led to the acceleration of the diabetic wound healing process.

### 6.3. Impaired Growth Factors Production

Growth factors play a critical role in initiating and sustaining the different phases of wound healing [120]. Several growth factors that are released at the wound site are presumed to be necessary for wound healing such as transforming growth factor (TGF), epidermal growth factor (EGF), fibroblast growth factor (FGF), insulin-like growth factor (IGF), keratinocyte growth factor (KGF), platelet-derived growth factor (PDGF). and vascular endothelial growth factor (VEGF) [120]. The down-regulation of growth factor receptors and rapid degradation of growth factors leads to delayed wound healing in diabetics [121]. TGF-β recruits and promotes the stimulation of inflammatory cells including neutrophils, macrophages, and lymphocytes, as well as keratinocytes, fibroblasts, and induces the production of growth factors [122]. The reduced concentration of TGF-β has been reported in diabetic wounds [123]. EGF is associated with the systemic attenuation of pro-inflammatory markers and antioxidant effects in diabetic foot ulcer patients [124]. Moreover, EGF stimulates fibroblast replication, collagen formation, and re-epithelialization which promote diabetic wound healing [125]. Lack of FGF-7 [126] and KGF [127] inhibited cell proliferation and delayed diabetic wound healing.

Some herbal products and their active constituents enhance cell proliferation, migration, and diabetic wound contraction via the stimulation of growth factors. *Momordica charantia* accelerated wound closure rate through intense TGF-β expression [40]. *Aloe vera*gel and *Teucrium polium* extract in an ointment significantly increased expression of VEGF, IGF-1, GLUT-1, and FGF-2 [24]. *Stryphnodendron adstringens* gel [50], arnebin-1 from *Arnebia euchroma* [19], and 20(S)-protopanaxadiol from *Panax notoginseng* [17] accelerated wound closure and elevated VEGF expression. Vicenin-2 facilitated healing in hyperglycemic conditions by increasing the release of growth factors such as VEGF and TGF-β to enhance cell proliferation, migration, and wound contraction via the VEGF and TGF-β pathways [71].

### 6.4. Impaired Keratinocyte and Fibroblast Proliferation and Migration

During the proliferative phase of wound healing, keratinocytes (epidermal skin cells), endothelial cells (the primary vascular cell type), and fibroblasts (the primary cell type in connective tissues) proliferate, migrate, and differentiate, which enables the formation of granulation tissue, reconstitution of the dermal matrix, restoration of surface integrity, and promotion of wound closure [128]. These processes may be supported by herbal products and their active constituents. Ointments containing *Aloe vera* or *Teucrium polium* alone and in combination triggered diabetic wound healing through fibroblast proliferation and collagen deposition [24]. Topical application of *Hypericum perforatum* in olive oil showed significantly higher tensile strength, tissue hydroxyproline concentration, and collagen density compared to the control group [36]. *Hypericum perforatum* gel improved tissue regeneration by enhancing fibroblast proliferation and collagen synthesis [37]. *Camellia sinensis* extract increased collagen and fibronectin deposition [31]. *Blechnum orientale hydrogel* exhibited re-epithelialization and higher fibroblast proliferation and collagen synthesis [61]. Curcumin from *Curcuma longa* loaded into electrospun nanofibers stimulated wound closure with well-formed granulation tissue areas dominated by fibroblast proliferation, collagen deposition, rapidly regenerated epithelial layer, and formation of sweat glands and hair follicle tissues [63]. Polysaccharides from *Astragali Radix* loaded into tissue engineering scaffolds increased collagen synthesis, wound closure, and appendage and epidermal differentiation [67].

### 6.5. Impaired Angiogenesis

Angiogenesis (or neovascularization) is an essential part of the wound healing process consisting of the formation of a new capillary network (microvasculature) in response to hypoxia or other stimuli [129]. The hypoxic conditions in diabetes induce macrophages to secrete pro-angiogenic growth factors such as FGF, VEGF, and PDGF and cytokines, such as TGF-β and IL-1 that are involved in the control of various aspects of angiogenesis [130,131]. VEGF is one of the most important angiogenic factors in wounds and its production lies downstream of hypoxia and hyperglycemia. Hypoxia following injury activates hypoxia-inducible factor-1 (HIF-1), a transcriptional activator that promotes angiogenesis by upregulating target genes such VEGF-A [132], while hyperglycemia induces indirect VEGF overexpression mediated by TGF-β [133]. In addition, the upregulation of FGF and PDGF is associated with angiogenesis in diabetes and stimulates wound healing in diabetic mice [134].

It is well known that chronic, non-healing diabetic wounds are closely linked to poor vascular networks. Herbal products and their active constituents are a rich source of novel angio-modulators that may affect the angiogenesis process in diabetic wound healing [135]. *Camellia sinensis* extract promotes the angiogenesis process and vascular remodeling via molecular control of circulating hypoxia-responsive microRNAs: miR-424, miR-210, miR-199a, and miR-21 in diabetic and non-diabetic wounds [31]. Topical application of the aqueous fraction of *Moringa oleifera* enhanced wound healing in diabetic rats through upregulation of VEGF and accelerating the angiogenesis process [41]. *Blechnum orientale* hydrogels [61], *Hypericum perforatum* gels [37], *Malva sylvestris* extract nanofibers [66], *Salvia kronenburgii* and *Salvia euphratica* ointments [48], a mixture of *Agrimonia eupatoria*, *Nelumbo nucifera*, *Boswellia carteri,* and pollen from *Typhae angustifoliae* [119] improved tissue regeneration by revascularization. 20(S)-protopanaxadiol from *Panax notoginseng* accelerated wound closure through elevation of VEGF expression and capillary formation, and stimulation of angiogenesis via HIF-1α-mediated VEGF expression by activating p70S6K through PI3K/Akt/mTOR and Raf/MEK/ERK signaling cascades [17]. Arnebin-1 from *Arnebia euchroma* in an ointment promoted wound healing by a remarkable degree due to neovascularization through the synergetic effects of arnebin-1 and VEGF [19]. Hydroxysafflor yellow A from *Carthamus tinctorius* and deferoxamine loaded in chitosan/gelatin hydrogels exerted a synergistic effect on enhancing angiogenesis by upregulation of HIF-1α expression [65]. Polysaccharides from *Astragali Radix* loaded onto tissue engineering scaffolds restored skin microcirculation [67], while polysaccharides from *Periplaneta americana* loaded in hydrogels effectively accelerated wound healing through inflammation alleviation, angiogenesis, and macrophage polarization [69]. Kirenol from *Siegesbeckia orientalis* [53], pongamol and the flavonoid-rich fraction from *Tephrosia purpurea in an* ointment [57], and curcumin from *Curcuma longa* loaded onto electrospun nanofibers [63] affected vascularization and angiogenesis in diabetic wounds.

## 7. Conclusions

There are numerous animal-based studies and only a few clinical trials confirming the activity of herbal products and their active constituents in the stimulation of diabetic wound healing. Topical applications of herbal products and their active constituents in formulation or loaded in various dressings seem to be a good alternative for the treatment of diabetic wounds. The new dressings offer several beneficial properties, such as absorbing excess discharge from the wound, maintaining a moist environment conducive to healing, creating a protective barrier against bacterial penetration, being suitable for wounds with necrosis, and affecting the release of active ingredients over time, which can positively affect diabetic wound healing. Herbal products and their active constituents through different mechanisms of action, including antimicrobial, anti-inflammatory, and antioxidant activities, stimulation of angiogenesis and keratinocytes, production of cytokines and growth factors, and promotion of fibroblast migration and proliferation, may be considered as an important support during conventional therapy or even as a substitute for synthetic drugs used for diabetic wounds treatment.

## Data Availability

The data presented in this study are available in this article.
